# Clinical Report on Dropsies

**Published:** 1819-07-01

**Authors:** 


*Clinical Report on Dropsies.* By John Crampton, M.D.

[Transactions of the Irish Colleges, Vol. II.]

Dropsy appears to be a disease of frequent occurrence in Dublin, and the greater number of those affected with such complaints resort to Steevens' Hospital; in the chronic wards of which, an excellent opportunity is offered for investigating a disease, to the pathology of which little has been added until within these very few years. It is unfortunate, indeed, that medical writers, in general, publish only the successful cases which they meet with—such, for instance, as correspond with their theories, or suit their practical views; whereas, it is only from the experience of a great number, and from an impartial account of *all*, the fatal as well as the successful cases, that any thing decisive can be collected.

This Report, embracing the compass of one year, contains a brief account of seventy-four dropsical patients. The first part gives the history and *post mortem* appearances of the fatal cases. The dissections are fifteen in number; from which an attempt is made to show what organs and textures are involved in the destructive changes. Six fatal cases could not be examined.

The second part of this Report gives the histories of fifty-three cases variously treated; thirty-five were, to all appearances, cured. In seven instances, considerable relief was afforded; some of them being altogether, others nearly emptied of dropsical accumulation. In eight cases, the patients left the hospital in *statu quo;* others, whose states were hopeless, averse to undergo further remedial trials, and anxious to return to their families, went away in progress to the ultimate termination. Three left the hospital suddenly, some of them for irregularity of conduct.

Of the thirty-five patients cured, twenty-three were subjected to general blood-letting, some of them four or five times, besides local bleeding and blisters; few of these took much inward medicines.

"There is no claim to novelty, but an anxious desire to ascertain what is true—to confirm what has been already observed, and to put remedies long in use to the rigid test of experience." 145.

Case 1. Mary C. ætat. 22, was admitted (27th of June) with general anarsarca; abdomen much distended, with evident fluctuation; pulse small and oppressed; respiration hurried; urine high coloured; excessive pain in the scrobiculus cordis. Dropsical symptoms of six weeks duration. Her husband had given her oxym. hyd. in solution, which was followed by severe pain in the bowels, suppression of the catamenia, and the dropsical swellings.

*M. M.* Bled twice at a few days' intervals, to the extent of ten ounces, which afforded temporary relief to the pain. The anasarcous swellings of the upper extremities subsided, and there was a considerable diminution in the size of the abdomen. Difficulty of breathing; return of pain in the scrobiculus cordis, greenish vomiting and hiccup came on, and she died in agony on the 8th of July.

*Dissection.* Lower extremities anasarcous; only two quarts of fluid in the abdomen; abdominal viscera sound; lungs healthy, though in some places adherent; more than half a pint of serous fluid, with flakes of lymph, in the pericardium; a white spot on the right ventricle, evidently from a deposition of coagulable lymph.

*Remarks.* This appears to have been primary inflammation of the inner surface of the pericardium, followed by dropsy of that bag, and consecutive effusion into the abdomen and cellular substance of the body. It is not improbable, that the train of morbid occurrences lay in the following order:—First, irritation in the stomach from the acrid mercurial; amenorrhœa from the same cause; next, congestion and inflammation in the membranes of the heart and pericardium, in a person predisposed; lastly, effusion into the different cavities and cells.

Had general and local detractions of blood been practised at an earlier period, when the local pain and the menstrual suppression pointed out that a new determination of blood had taken place, and, probably, that inflammation had commenced in an organ of the first importance, the result might have been different.

Case 2. E. A. ætat. 19, July 4th, 1817. Anasarca of the face, head and arms, legs, thighs, and body, has come on in the order of the parts enumerated. Abdomen distended, and fluctuates; lips and cheeks purple and livid; cough and pain of the left side; pulse small and indistinct; urine scanty and red; diarrhœa. He has been dropsical for seven months: it came on immediately after a fall from a car.

He was partially relieved by a single venesection, the cough and pain of his side having been removed. In other respects the treatment was ineffectual. He died on the 18th.

*Dissection.* Body anasarcous; little fluid in the abdomen; liver enlarged, presenting a peculiar marbled appearance; other abdominal viscera sound, as were the lungs; heart adherent to the pericardium in many points, by bands not of recent formation.

*Remarks.* Dropsy frequently follows an injury sustained by a fall, as in this instance; in which cases, the irremediable changes of structure generally take place in the heart, liver, or lungs. In the present instance, the disease evidently originated in inflammatory affections, chiefly in the cavity of the pericardium and in the liver. Here, as in the first case, anasarca of the face may be noticed as a symptom very frequently attending disease of the heart. Dr. Crampton thinks it may be considered as one of the diagnostics of hydro-pericardium.

Case 3. John M'Dermot, ætat. 32, (January 26, 1818,) was seized with shivering, after exposure to cold, about a fortnight ago, followed by cough, hoarseness, pain of chest, dyspnœa, and oppression. To these succeeded general anasarca and ascites, with frequent pulse and high-coloured urine. Venesection to ten ounces, which was repeated on the 29th; his cough, pectoral distress, and hoarseness having continued. His swellings are now diminished.—2d. February. After injudicious exposure to cold frosty air, he was seized with shivering; acute pain in the chest; increased cough and dyspnœa. Blooded on the 2d and again on the 3d.—On the 16th of February, his dropsical swellings were gone; but the continuance of dyspnœa, irregular pulse, and strong pulsation in *epigastrio*, clearly indicated a cardiac affection of momentous import. He died on the 1st of March.

*Dissection.* Lungs sound; heart thrice its natural size, the increase of volume being confined to the left ventricle; aortic semilunar valves "converted into a polypus-like substance, mixed with ossific matter."—A similar diseased appearance within the ventricular valves, where were also excrescences about the size of garden peas. Liver much enlarged, but the structure not sensibly altered; spleen greatly enlarged, and, in structure, softer than natural.

*Remarks.* This case shews, that although medical treatment was resorted to, too late, yet that the bleedings employed for the relief of the thoracic symptoms, did not interrupt the removal of the dropsical effusions; on the contrary, they appeared to expedite their departure. It is also probable, that although organic derangement might have been going on in the heart long prior to the date of his present illness, yet that the dropsy was not established till exposure to cold induced an inflammatory state of the circulating organ, and accelerated the march of disorganization in the valvular apparatus. The liver and spleen were merely in a state of congestion, which would have been easily removed by detractions of blood and purgatives, had the pectoral maladies admitted of a prolongation of his life.

CASE 4. Bridget Harper, ætat. 30, (May 1817) has been four months dropsical. General anasarca; fluctuation in the abdomen; pale and leuco-phlegmatic. Says the disease came on with cough and dyspnœa; pains in the shoulders and across the clavicles; high-coloured urine. She soon died, under the treatment of diuretics and light cordials.

*Dissection.* Lungs sound; half a pint of water in the pericardium; a white spot, the size of a shilling, on the heart, apparently a deposit of coagulable lymph. Four quarts of straw-coloured fluid in the abdomen; liver slightly tuberculated, with somewhat rough surface.

The deposit of coagulable lymph, Dr. C. observes, on the surface of the heart, and the effusion of fluid into the pericardium, after the inflammatory symptoms with which the disease commenced, plainly shew what was the original character of her complaints. In this instance again, anasarca of the face was associated with disease of the heart. We have often observed this symptom ourselves, and are at this moment attending a gentleman of high rank in life, and a most worthy member of society, who has unequivocally disease of the heart, attended with this anasarcous state of the face, especially of the right side

In this case, dropsical effusions in the cellular substance of the lower extremities, attended with symptoms of hydrothorax, have been repeatedly removed by a combination of squill, blue pill, and digitalis; but the original cause, which we conceive to be vulvular disorganization of the heart, of course remains, and obliges us to renew our means every month or six weeks.

CASE 5. C. R. ætat. 30, (June 30, 1817), a pale, livid, emaciated-looking man, intemperate, and suffering from cough and dyspnœa for two years, became suddenly anasarcous in his legs, thighs, and scrotum, with distended abdomen; small pulse, and scanty high-coloured urine. His treatment consisted in a blister to the chest, diuretics, and mild aperients. The abdominal swelling was reduced; the scrotal œdema disappeared, but his respiration was not relieved, and he died on the 6th of July.

*Dissection.* Limbs anasarcous; abdominal viscera healthy; liver turgid with blood; lungs adherent to costal pleura on both sides, heavy, and studded throughout with small tubercles, some of them suppurating. Some fluid *in pericardio*; vessels of the heart loaded with blood.

*Remarks.* In this case, the lungs and their investing membranes were in fault; the liver in a state of venous congestion, probably from the man's habits of intemperance.

CASE 6. Lucy Swift, ætat. 28, (July 25th, 1817) has been generally anasarcous for eight months prior to her admission. Ascites is present. The first dropsical symptom was œdema of the face, accompanied by dyspnœa. She has an occasional purple tinge on the cheek, with leuco-phlegmatic looks. Pulse 80, hard, and cordy; urine scanty and red. Catamenia had been suppressed shortly before her breathing became affected. Ascribes her disease to lying in a cold damp cellar.

She had been bled twice before her admission. She was bled to ten ounces this day, chiefly at her own request; her pulse being cordy and irregular, her face flushed, with a circumscribed spot.

Aug. 1. Pulse continued hard; cough incessant; strong pulsation of the carotids. Another bleeding relieved the urgency of the symptoms, and the swellings were diminished; but her dyspnœa and decubitus difficilis increased, and she died on the 18th of August.

*Dissection.* Considerable serous effusion in the abdomen; coats of the intestines thicker than natural; mesenteric glands enlarged; liver enlarged, gorged with blood, and beginning to undergo organic changes on its surface.

On raising the sternum, no lungs could be discovered, being hidden by the heart and its membranes; a pint of fluid *in pericardio.* Heart thrice its natural volume; parietes of the *right* ventricle softer and thinner than natural; the increase of thickness was in the left ventricle.

*Remarks.* Cold and damp, concurring with suppression of the catamenia, were causes adequate to the production of an inflammatory irritation in the thoracic organs. Why the disease fell on the heart and its membranes, in preference to the lungs, can only be explained by a greater local predisposition to morbid action in the former than in the latter viscus. Had this distressing case been met by a vigorous depletory practice on the occurrence of the menstrual suppression, the press of blood on the heart, and consequent enlargement of that organ might have been prevented, as well as the subsequent effusion into the pericardium.

CASES 7 *and* 8. In the first of these cases, the liver was in a state of great disorganization, and consisted almost wholly of tubera circumscripta, while the lungs were tuberculated throughout, and adherent to the pleura costalis on the right side. In the second case, the thoracic organs were sound, but the liver was covered with tubera circumscripta. The dropsical effusions were of course merely symptomatic, and dependent on the morbid state of the liver in both instances.

Dr. Crampton here observes, that the scanty urine with high red sediment, which tinges linen, was seldom wanting in cases of scirrhus liver, differing in *colour* from that tinged with bile, and in *intensity* from the usual deposits in the other forms of dropsy The pallid, dusky, emaciated appearance, with shiverings and profuse sweats, are, for the most part, demonstrative of this form of the disease.

CASE 10. [Case 9 passed over]. William Tuite, ætat. 24, a newsman, November 10th, 1817, was admitted with relapse of general dropsy, both anasarca and ascites, to a considerable extent. Breathing much distressed; pulse hurried; urine scanty and red; no cough nor local pain. He had been cured of dropsy in September of the same year; but returning to his intemperate habits, and being constantly exposed to cold and wet, the disease recurred.

He died on the 19th of December, having suffered severe pain in the umbilical region, and excessive abdominal distention for some days antecedent to his death.

*Dissection.* Abdomen enormously distended with fluid. On opening it, a portion of the omentum was found adhering by recent exudations to the umbilicus. Liver diminished in size, but tuberculated throughout. Its peritoneal covering coated with coagulable lymph, as was the peritoneal lining of the abdomen. Thoracic viscera in a sound state.

*Remarks.* Tuite's case is very interesting, as he was twice subjected to medical treatment within a short space of time. In the first instance, as will be seen in a future part of this article, his attack of dropsy was met with repeated venesection, blisters, and other antiphlogistic remedies; and the result was a complete cure. In the second instance, though his disease did not appear more formidable, the result was unfavourable. The same active treatment was *not* adopted. Too much reliance was placed on cathartics and diuretics. The tuberculated liver, undoubtedly in itself, was sufficient ultimately to undermine his health; but his lungs were good, and with cautious treatment and temperate habits, his life might have been prolonged for a considerable time.

CASE 11. T. W. ætat. 60, (June 9th, 1817) had had anasarca in the legs, and cough for upwards of a month; his abdomen swelled, hard, and tense during the last fortnight; urine scanty and red; disease attributed to cold.

Venesection was practised twice in this case, without any benefit to the cough. Blisters, blue pill, and cream of tartar, were also ineffectual. He died on the 27th of June.

*Dissection.* A gallon of serum in the abdomen. Peritoneum inflamed, and its surface covering the intestines coated with a layer of lymph, especially in the epigastrium, and on the surface of the liver. This organ studded with small tubercles. The spleen double its usual size, and its surface studded with tubercles. Coats of the intestines thicker than natural, from effusion of lymph between their laminæ. Lungs sound, free from adhesion; aortic valves ossified.

*Remarks.* A complication of causes here conspired to give rise to the general dropsical state. Tuberculated liver, disease in the aortic valves, enlargement of the spleen.—

All these changes of structure must have existed long before the effusion into the cavity of the abdomen; but yet they do not seem to have completed the hydropic disease, until a general inflammatory affection of the peritoneum took place; and cold was the exciting cause.

Case 12. James Lawler, ætat. 15, (June 15th, 1817). General anasarca, and considerable ascites of three months' date; cough, with mucous expectoration for four years; breathing distressed; lips livid; face pale, with a circumscribed purple spot on each cheek; pulse frequent, small, and irregular; urine scanty and high coloured; pains occasionally in the chest. His treatment consisted in the use of mild aperients, with diuretics, calomel, and blisters. The anasarcous swellings were considerably reduced before his death, which took place on the 9th of July.

*Dissection.* Three pints of serous fluid, with flakes of coagulable lymph diffused through it, in the cavity of the abdomen. Marks of inflammation were also observable on many parts of the membranes in this cavity, particularly on the surface of the spleen, and concave surface of the liver. Lungs sound; apex of the heart connected to the pericardium by a membranous band; many spots on the surface of the heart resembling petechiæ.

*Remarks.* It may appear extraordinary that cough should have existed so long, without the lungs being affected in structure. The distention of the abdomen and the condition of the heart, however, were quite sufficient to explain the dyspnœa and cough; and it is not improbable, that had the mucous membrane lining the bronchi been minutely examined, other causes for the long subsistence of the catarrhal state, would have been discovered. A chronic inflammatory affection of the heart and its membranes had probably existed for a considerable time, effusion perhaps being prevented by a vicarious discharge from the bronchial surfaces. It was not until a more acute inflammatory disease had occurred in the membranes of the abdomen, that the dropsical effusions became established.

---

From the detail of the preceding cases, it may fairly be inferred that, at least *in many* dropsical diseases, inflammation of some organ or texture either precedes, in form of cause, or supervenes in the course of the malady, as a material aggravation of the affection, and obstacle to the practitioner's plans of treatment.

In many instances, Dr. Crampton thinks, in incipient dropsies, sanguineous congestion in the lungs, liver, spleen and other organs is present, especially in the *venous* system of these viscera, leading ultimately to effusion, unless purgatives, with abstemious diet, be prescribed. If the congestion obtain in the lungs, venesection may be advisable, although no actual inflammation be present; for it is very necessary to relieve that important organ, and prevent the habit of a serous secretion into the bronchial tubes, by which humoral asthma may be established, and dropsy ultimately result. If this congestive state is allowed to subsist, and medical treatment be neglected, inflammation will supervene from mere vascular distention; effusions of serum and lymph will follow; adhesions and false membranes will be formed on the surface of the serous coats, and thus additional difficulties are thrown in the way of the physician. In the first instance, he has only a disease of *function* to correct; in the second, a disease of structure to contend with.

We have now presented our readers with an analytical view of the *pathological* part of Dr. Crampton's valuable Report; or that containing the fatal cases, with the appearances on dissection. The Clinical Report of cases *successfully* treated, forms quite a distinct Report, which we shall now proceed to analyze.

Dr. Crampton justly observes, that in attempting the relief or cure of Dropsy, the *early* symptoms should be strictly attended to and ascertained, in order that we may be able to recognize the organ primarily attacked, and the disorganization that may be impending; for after the disease has become generally extended, it is difficult to calculate on the condition of the principal viscera. The stage of the disease is likewise to be taken into consideration, since remedies which are useful at an early, may be injurious at a more advanced period.

From the result of the cases by Dr. Crampton, it appears that a greater number of dropsies, connected with disease of the thoracic viscera, were relieved by medicines, or cured, than where the viscera of the abdomen were concerned. Ten of the fifteen patients examined after death had either the liver, stomach, or spleen tuberculated; combinations which appear more formidable than even hydrothorax, combined with organic disorder in the cavity of the chest, provided the organic derangement is at all compatible with the functions of circulation and respiration.

Of the patients cured, a considerably greater proportion were affected with disease in the thoracic viscera; some of them evidently organic affections of the heart; and yet they appeared to be acted upon by remedies with infinitely more ease, than where the disease had established itself in the cavity of the abdomen. We should not, therefore, be too confident of success in ascites; nor despair in hydrothorax, even where the pulse is feeble and intermitting; the breathing difficult and laborious.

The result of our author's experience warrants him in stating that, whenever the organs of respiration appear to labour, the strength not being much impaired, and the stage of the disease not being far advanced, general bleeding may be safely practised; particularly if there exist, in addition, any symptoms indicative of inflammatory action in any texture within the thorax. In some cases, a single venesection appeared to arrest the progress of a recent dropsical disease; in others, a repetition of the measure was necessary to ensure success. In these complications, other remedies, without blood-letting, were useless. Diuretics would not act; purgatives afforded no relief till after the sanguineous evacuation.

When the congestion or inflammation is removed, it is less difficult to regulate the secretions. When dropsy is maintained by an acute or subacute inflammatory condition of the thoracic viscera, masked by debility, and not developing itself by legitimate symptoms, venesection will often disclose the true state of the case, in the quality of the blood. In incipient dropsy it is generally buffed, but not always;—at all events, a small venesection can do no harm, if cautiously practised. In advanced stages of dropsy, the blood drawn shews milky serum; crassamentum small in quantity, but often cupped, as in the blood of diabetic patients. This appearance of the blood is often observed in dropsy connected with tuberculated liver. In such cases, at such a period, general bleeding is usually injurious, unless recent signs of inflammation have been superadded to those already in existence. Here local detractions of blood, followed by blisters, are more effectual. And these remarks are particularly applicable to ascites in debilitated subjects, with an inflamed state of the peritoneum. Mild aperient medicines to regulate the discharges from the bowels are quite sufficient after such preparatory discipline. The following case, from private practice, is given as an exemplification of this point of treatment.

Miss H. ætat. 14, a delicate girl, much-emaciated, fell suddenly into ascites, with a tense hardness in the epigastrium; pulse 120; skin dry; tongue white; urine scanty and high coloured, with red sediment. Dropsy of a fortnight's duration, preceded by chilliness, and ushered in with thirst, languor, and inappetency. She had been affected with chorea a year before, for which cinchona, steel, and port wine, had been prescribed. Subsequently she experienced pain in both sides, for which blisters were advantageously applied. Her feeble and apparently hectic state forbade the lancet, though it was evident that a chronic inflammatory condition had existed for some time. Twelve leeches to the epigastrium; ten more to the lower abdomen, the following day, which procedure was repeated four times on alternate days, a warm bath being directed every third night. Internally, two grains of calomel and a drachm of supertartrite of potash were daily exhibited for a week, when the calomel was omitted, and a draught, with infus. rhei. and chamomile, was substituted. In a fortnight, the dropsy was gone; the urinary secretion restored; and in three weeks she had began to recover flesh. P. 263.

Although in dropsical affections, which are symptomatic of confirmed phthisis, general bleeding is very prejudicial, yet in such affections from chronic inflammation of the pleura, of the bronchia, or of the parenchymatous structure of the lungs, venesection is frequently the means of rapidly restoring the patient to health.

Where the liver is concerned in dropsy, much will depend whether the disease be of function or of structure. If there be reason to apprehend vascular plethora, or venous congestion of that organ, or inflammation of its membranes, blood-letting will expedite the cure; and still more so if unequivocal symptoms of hepatitis obtain. The more recent the case, the better the opportunity for general sanguineous depletion; in more advanced periods, repeated leeching and cupping, followed by blisters, promise more. Purgatives, diuretics, and mercurials, will act with greater advantage after such preliminary treatment, and considerably smaller doses will answer.

" If our knowledge of diagnostics enables us to ascertain those cases of dropsy which are complicated with tuberculated liver, spleen, pancreas, or ovarium, it should make us abstain from the use of the lancet, more especially where there is reason to apprehend a general tubercular diathesis. Local bleeding is sometimes applicable to such cases, when the peritoneum covering the tuberculated liver, spleen, or pancreas, becomes inflamed." 265.

We fear there is yet nothing like a diagnosis between tuberculated liver and many other disorganizations of that viscus. There is no form of dropsy in which detractions of blood are more useful than those where the peritoneum is inflamed, and where ascites follows. Local bleeding, after general venesection, frequently removes the inflammatory state of this membrane, and leaves little for the accomplishment of other remedies. It is not difficult to distinguish these latter cases from those attending a tuberculated liver. In dropsy arising from inflamed serous membranes, the pains are superficial, and felt on pressure. Those from scirrhous liver are deeper seated; the general health more broken up; the frame more emaciated; and the urinary sediment of a deeper red. When its attacks are sudden, after exposure to cold, venesection is generally adviseable; When its approach is more gradual, after abuse in spirituous liquors, there is more reason to suspect schirrhus, and the treatment must be more guarded.

Ascites is not a very uncommon sequela of dysentery; in which cases, the inflammation of the mucous membrane has spread to the serous coverings of the intestines. General venesection, where this conversion or extension of disease is recent, and leeching or cupping, where the disease is chronic, will generally arrest the dropsical effusion; "and if the textures concerned are not too deeply involved in destructive changes, mild mercurials with opium and gentle purgatives, will, for the most part, complete the cure."

The same observations apply to those dropsies which follow the puerperal state. These are almost always connected with an inflamed condition of the peritoneal membranes, and general or local bleeding may be employed with more freedom, the more recent the attack. Amenorrhœa is frequently the forerunner of dropsy, as well as a concomitant of it. If sanguineous depletion is early and freely used on the appearance of congestion in any important viscus, dropsical effusion will rarely follow; even when it has taken place, an active treatment of this kind will soon make the swellings disappear.

"There is not much to be inferred from the preceding report, as to the comparative efficacy of digitalis, squill, colchicum, cream of tartar, and other remedies usually employed in dropsical diseases. Each of these has succeeded where the patient was properly prepared for their employment; but it appears plainly that none of them will prove effectual if they are prescribed too early, nor can we rely solely on them." 267.

A combination of squill and digitalis was employed oftener than any other medicine by Dr. Crampton, though colchicum was found a useful and active diuretic where squill or digitalis had disagreed. In many instances the powdered leaf of elaterium proved a useful adjuvant in depleting dropsical patients. Elaterium, he thinks, will be found to answer better in those dropsies connected with disease in the serous membranes of the abdomen, where there is torpor of the mucous coats.

"I need not state how very general the use of mercury has become in this class of diseases. In the greater number of incipient dropsies, I believe, it not only fails, but often aggravates the symptoms, by adding to the excitement, and increasing the inflammatory disposition. But in more advanced periods, and even earlier, after timely venesection and other preparatory expedients, mercury proves a remedial agent of no inconsiderable efficacy." 270.

The plan of treatment, our author observes, where early venesection in dropsical diseases is recommended, must appear very abhorrent to those who were accustomed to consider the dropsical or serous diathesis as the result of atony or weakness. Relaxation in the exhalent system is still considered one of the principal causes of dropsy; and this doctrine we know is still taught by the majority of our metropolitan lecturers.

"Whereas those who look to the diseased appearances in the different cavities, are more disposed to conclude dropsy as associated with an excited condition of the exhalents pressed by the *vis a tergo* of the capillaries, and oozing out their fluids more especially on the serous membranes, which are so constructed as not to allow the same distention of their vessels which other textures permit." 271.

Well may Dr. Crampton say that the very name of dropsy, and the notions of debility and relaxation have too long tied up the hands of practitioners; and that it is high time these delusive theories should give place to facts, and reasonings founded on these facts.

"It would be well, therefore, in forming our plans of treatment, to lose sight of the name of dropsy, and take measures to prevent those organic changes which we are apprehensive are going on. Nosology, in giving systematic names to diseases, has facilitated the study of medicine; but inclines us to dwell too much on symptoms, and too little on the real pathological state." 272.

In a considerable number of the cases, of which we shall presently give some account, the urine was tried by the test of heat, as to its powers of coagulation; but the proportion of instances where it took place was very inconsiderable, compared with those where it did; nor was Dr. Crampton able to connect those cases where inflammatory symptoms existed with the presence of coagulable urine. "In many of those which appeared to require the prompt use of the lancet, the urine did *not* coagulate." Under this impression, he ceased to draw any practical inference from the appearance of the urine, and discontinued making any further experiments on the subject.

"Dr. Edward Percival, who was my predecessor as one of the physicians to the House of Industry, mentions that the result of his experience on this subject fully coincides with mine. After he had tried dropsical urine by the test of coagulation in a number of cases, he at length lost all confidence in the test, either as an invariable evidence of inflammation, or as a guide of practice. His statement is confirmed by the additional testimony of Dr. Reid, who acted as clinical clerk at the House of Industry, at the time these experiments were made." 274.

We shall now proceed to select and condense a few of the numerous cases brought forward by Dr. Crampton, in elucidation of the treatment of dropsy.

Case 1. June 6, 1817, J. Bishop, ætat. 24, a stout, muscular man, affected with anasarca of the legs, thighs, scrotum, and cellular membrane; abdomen considerably distended, with evident fluctuation; head-ache; cough; pain of the back and belly; pulse 80; urine scanty, and high coloured. Swellings of a fortnight's duration, and attributed to cold. Venesection to twelve ounces; opening electuary; blood buffy; and he experienced relief of cough and pain. On the 9th, the swelling of the scrotum disappeared; abdomen slacker; legs less swelled; urine more abundant, and less loaded; venesection repeated to ten ounces. 13th. All swellings gone. From this to the 20th complained only of a slight cough, and a sensation of soreness in the lower belly. Small doses of pil. hyd. and an aperient mixture were directed; and as the epigastric region felt hard and tender to the touch, ten ounces of blood were taken from the arm, and a large blister applied over the epigastrium. All uneasiness was removed by the 27th, and he was discharged cured.

Case 2. July 11, 1817, J. D. ætat. 34, a sailor, a strong muscular man, with considerable ascites, and very general anasarca of six months date; affected also with cough; pain in his chest; full, hard pulse; pale urine, uncoagulable by heat; disease attributed to cold, fatigue, and intemperance. Venesection to twelve ounces; purging electuary. On the 18th, although his cough was easier, pulse softer, and swellings diminished, venesection was repeated. Blood was buffy in both instances. A third bleeding was practised on the 21st, as he still complained of his chest. A fourth venesection on the 28th. He bore all these bleedings well, and expressed himself relieved each time. August 4. The anasarca and ascites being much reduced, a hardness and tenderness were observed in the right hypochondrium. A blister applied, and pills of squill, calomel, and digitalis directed, with an occasional laxative. "His mouth became sore; the fullness and tenderness in the region of the liver went off; and he was discharged perfectly cured on the 8th of September."

*Observations.* In this case there was inflammation in some of the textures of the pulmonary apparatus; sanguineous congestion in the liver, its membranes probably inflamed, and the whole peritoneum exuding a serous fluid. These morbid processes were arrested by venesection; but full relief was not obtained till after the fifth detraction, amounting in all to fifty-two ounces. It may be noticed with what advantage mercurials act, in many diseases, *after* sanguineous depletion; indeed, their failure is often owing to a want of the proper preliminary treatment in this way. The same observation applies to digitalis and squill, as was long ago noticed by Withering, and is exemplified in the foregoing case.

Case 3. October 10, 1817, W. Murphy, æt. 23, a vigorous man; a fortnight before admission was seized with oppressed breathing, cough, hoarseness, and palpitation of the heart; his face became œdematous; his abdomen next swelled; and lastly, anasarca of the legs and thighs followed. Pulse 80; urine high coloured and scanty. Venesection to ten ounces, and an electuary with supert. potass. 13th. Twelve ounces more of blood were abstracted, with evident relief to the pectoral symptoms. Squill, digitalis, and calomel prescribed. 17th. A further venesection of ten ounces was practised, an increase of hoarseness and cough having occurred from fresh exposure to cold. Swellings much diminished. 27th. All dropsical symptoms disappeared; the respiration still impeded, but the urinary secretion restored; mouth slightly sore. 10th of November discharged perfectly cured.

*Observations.* Here a very formidable disease appears to have been subdued in a very short time. Dr Crampton conceives, and with great probability, that both the heart and lungs were the seat of subacute inflammation, which had given rise to effusion, and which, very likely, would have ended in change of structure in these organs, had not a very active treatment been employed.

CASE 4. Feb. 9, 1818. Owen M'Cabe, ætat. 50, labourer, a tall, sinewy man, is anasarcous all over, with ascites. He has cough, oppressed breathing, an intermitting pulse, scanty high coloured urine, and a circumscribed purple spot on each cheek. Dropsy has been present six weeks; cough and dyspnœa he has had a considerable time. Disease attributed to cold and hard labour. Venesection to ten ounces; a blister to the chest, and an aperient electuary. A second blister was applied to his back a few days afterwards. Pills of calomel, squills, and digitalis were also prescribed. On the 9th of March, the dropsical swellings were gone, except from his legs; the œdema shifted, from his lying in bed, to his right thigh, which became erysipelatous. At this time his palpitation was distressing; his pulse intermitting; and his strength much reduced. Infus. gentian. with acet. colchici ordered. March 30th. Erysipelas of the thigh has suppurated extensively. April 13th. Dropsy gone; erysipelas has disappeared; palpitation continues. May 10th, discharged cured.

*Observations.* The history of this case shews that dropsy was only symptomatic of the state of the pectoral organs. A chronic carditis appeared to accompany an aneurismal condition of the heart, and effusion was the result. The case was almost hopeless in the first instance, and still more so when the erysipelatous inflammation attacked the œdematous limbs. Erysipelas indeed is a frequent attendant on those dropsies dependent on an aneurismal state of the heart, and the result is almost uniformly fatal. In the present case there is every reason to fear that the dropsy will return on the reapplication of the occasional causes. We cannot say how far the powers of life will endure changes of structure in important organs; but we know, in many instances, that partial relief from medicine will often arrest the progress of destructive maladies. Hence, by palliative treatment, where a radical cure is impossible, life may be prolonged under the pressure of urgent diseases, and human suffering be much diminished; of which the case before us affords a most striking illustration.

CASE 5. May 23, 1817, William Tuite, ætat. 24, a newsman. [This is the man whose relapse, fatal termination, and dissection, are stated in the first part of this report, Case x.]

W. T. was a strong muscular man, addicted to the abuse of ardent spirits. He became affected with very general anasarca, seven months before his admission, together with abdominal ascites of unusual size, the abdomen being tender on pressure all round; cough, and pain of chest; urine scanty and red; has been exposed to severe weather. Venesection to twelve ounces; four grains of calomel and an aperient electuary ordered. 6th June. A second bleeding to ten ounces; blood buffy in both instances; chest relieved; tenderness of the abdomen removed. June 9. A third venesection; after which, the swellings were a little reduced. 13th. Œdema has left the legs; abdomen softer. 20th. Mouth sore; calomel omitted; tincture of squill and digitalis directed. 23d. Still further reduction of the swelling. 30th. Return of pain in the right side; a fourth bleeding to ten ounces. July 4. Anasarca gone; abdominal enlargement not quite subsided; blisters in succession to the parietes of the belly. 22d. Discharged cured.

*Observations.* Our readers are aware that Tuite became again dropsical, died, and was examined, as before related. This man was kept a long time in hospital, under the hope of weaning him from his baneful habits of inebriation; for it ought to be remembered that inflamed and altered textures require a considerable time to regain their healthy condition. In his second and last attack, there is reason to believe that a vigorous treatment of sanguineous depletion would have again conquered his disease; but the absence of cough and local pains in the abdomen decided the point against venesection, although it was afterwards found that he died chiefly from acute inflammation of the peritoneal coat of the omentum. This case is instructive, as to the pathology of dropsy, and throws no small light on the treatment of such diseases, when not of very old standing.

Case 6. October 10, 1817, Sarah Holt, ætat. 35, a pale, sallow woman; abdomen extremely large, tense, and sore on pressure; legs anasarcous; pulse small and frequent; urine scanty and high coloured. Disease came on with dysenteric symptoms, and blood in the stools; it has subsisted for a month. Ascribes the complaint to cold and bad food.

Medical treatment in the hospital, which lasted four months, consisted of four general bleedings; repeated cupping on different parts of the abdomen, according to the varying seat of the local pain; a succession of blisters to the same surface. Internal medicines, at first blue pill with opium, until the dysenteric symptoms were removed; afterwards, squill, digitalis, and colchicum, at different periods, with jalap and cream of tartar occasionally. She was discharged cured on the 21st of February, 1818.

*Observations.* Here the inflammatory action appears to have spread from the mucous to the serous membranes of the abdomen, with effusion as the result. Chronic peritonitis was, therefore, the chief disease, the dropsy being merely symptomatic. By subduing the phlegmasia, the increased secretion was checked, and the hydropic disorder removed.

CASE 7. February 20, 1818.—Catherine M'Cann, ætat. 24. Abdomen is enlarged, hard, and evidently contains a fluid; pulse rather frequent; tongue white; urine high coloured. Enlargement came on eighteen months ago, after a miscarriage, and was attended with severe pains in the abdomen at the time, which recurred occasionally since. Venesection to ten ounces; small doses of calomel; bowels regulated by castor oil. 23d. The pains gone; swelling has already begun to diminish, and the urinary secretion to increase, and become of a better colour. Ex. col. comp. with calomel every night, and an aperient senna mixture on alternate mornings. Under this treatment she recovered, and was discharged cured on the 9th of March.

*Observations.* The puerperal state frequently gives rise to inflammatory affections of the serous membranes of the abdomen. When these are acute, and attended with fever, the termination is too often fatal; when chronic, it is generally succeeded by effusion, unless suitable treatment is adopted.

We think that in disseminating the mass of important facts and excellent observations contained in Dr. Crampton's Report, among the minute ramifications of the Profession, we have "done the state some service." His views and practice so entirely coincide with our own, that we have been very sparing of any deviation from the strict line of analysis, in the present paper; and we cannot conclude without recommending the Report to the most serious consideration of every class of Medical Society, as a document fraught with intelligence, sound pathology, and successful practice.

